# Characterization of human FcεRIα chain expression and gene copy number in humanized rat basophilic leukaemia (RBL) reporter cell lines

**DOI:** 10.1371/journal.pone.0221034

**Published:** 2019-08-20

**Authors:** Eman Ali Ali, Marina Kalli, Daniel Wan, Ryosuke Nakamura, David Onion, Daniel G. W. Alanine, Marcos J. C. Alcocer, Franco H. Falcone

**Affiliations:** 1 Division of Molecular Therapeutics and Formulation, School of Pharmacy, University of Nottingham, Nottingham, United Kingdom; 2 School of Biosciences, University of Nottingham, Sutton Bonington Campus, Loughborough, United Kingdom; 3 National Institute of Health Sciences, Kawasaki, Japan; 4 School of Life Sciences, University of Nottingham, Nottingham, United Kingdom; University of Salerno, ITALY

## Abstract

Several laboratories have created rat basophil leukemia (RBL) cell lines stably transfected with the human high affinity IgE receptor (FcεRI_H_). More recently, humanized RBL cell lines saw the introduction of reporter genes such as luciferase (RS-ATL8) and DsRed (RBL NFAT-DsRed). These reporters are more sensitive than their parental non-reporter humanized RBL cell lines. However, no studies so far have addressed the levels of FcεRI_H_ surface expression on humanized RBL cell lines. This is a critical parameter, as it determines the ability of these cells to be efficiently sensitized with human IgE, hence it should affect the sensitivity of the cell assay–a critical parameter for any diagnostic application. Our purpose was to assess and compare the levels of expression of the transfected FcεRI_H_ chain in humanized RBL cell lines. We compared surface levels of FcεRIα_H_ by flow cytometry, using a fluorescently labelled monoclonal antibody (CRA-1/AER-37) and determined receptor numbers using calibration microspheres. FcεRIα_H_ copy numbers were assessed by qPCR, and the sequence verified. Transfection with FcεRIγ_H_ cDNA was assessed for its ability to increase FcεRIα_H_ expression in the NFAT-DsRed reporter. While both SX-38 and RS-ATL8 expressed about 500.000 receptors/cell, RBL 703–21 and NFAT-DsRed had approximately 10- to 30-fold lower FcεRIα_H_ expression, respectively. This was neither related to FcεRI_H_ gene copy numbers, nor to differences in steady state mRNA levels, as determined by qPCR and RT-qPCR, respectively. Instead, FcεRIα_H_ surface expression appeared to correlate with the co-expression of FcεRIγ_H_. Stable transfection of NFAT-DsRed cells with pBJ1 neo-huFcεRI gamma, which constitutively expresses FcεRIγ_H_, increased FcεRIα_H_ chain expression levels. Levels of FcεRIα_H_ surface expression vary greatly between humanized RBL reporter cell lines. This difference will affect the sensitivity of the reporter system when used for diagnostic purposes.

## Introduction

Humanized rat basophilic leukaemia (RBL) cell lines derived from the parental RBL-2H3 cell line [1,2] are increasingly used for detection of allergen-specific Immunoglobulin E (IgE) in human blood samples [3]. As a minimum requirement, these cell lines need to be stably transfected with the human FcεRIα (FcεRIα_H_) chain, as the rat homologue receptor does not bind human IgE with high affinity [4]. Therefore, in order to assess human sensitization, several groups have created stably transfected humanized RBL cell lines, such as RBL SX-38 [5], RBL 48 [6], RBL 703/21 (which was derived from RBL 30/25 [7]), or RBL hEIa-2B121 [8]. Similarly, RBL-2H3 cell lines have been transfected with non-human receptors allowing allergen-specific IgE measurements in canine [9] and equine [10] blood samples.

The most recent generation of transgenic cell lines has further modified these humanized RBL lines to include sensitive and easy to measure reporter genes, such as firefly luciferase (RS-ATL8; [11]) or red fluorescent protein (NFAT-DsRed; [12]). These reporter cell lines have a series of advantages over the older generation, which we have described in detail in a recent review [3].

RS-ATL8 cells (EXiLE [11]) have been used for assessment of allergenicity of parasitic antigens [13] and for elucidation of complex sensitization patterns in individuals who had been sensitized to ingested wheat after using a soap-product containing acid-hydrolysed wheat protein [14]. However, despite their proven usefulness, there is only scarce information regarding the surface expression levels of the transgenic alpha FcεRIα_H_ chain across these humanized cell lines. Furthermore, the stability and extent (i.e. gene copy number) of transgene integration has not been reported. These are critical parameters, as they determine the ability of these cell lines to efficiently bind human IgE in serum samples used for sensitization, and therefore define the lower threshold of detection i.e. sensitivity, in addition to the method used for detection of activation (e.g. luciferase vs. beta-hexosaminidase assay).

Here, we assess and compare gene copy numbers of FcεRIα_H_ chain as well as surface expression levels in SX-38, RS-ATL8, RBL-703/21 and NFAT-DsRed cell lines, and discuss the significance of our findings in the context of human IgE measurements.

## Materials and methods

### Cells

RBL-2H3 cells were obtained from the European Collection of Cell Cultures (ECACC), UK (Catalogue No.: 86061001). RBL-703/21 cells were provided through a Material Transfer Agreement (MTA) by Stefan Vieths and Lothar Vogel (Paul-Ehrlich Institut, Langen, Germany). RBL SX-38 cells were provided through an MTA by Jean-Pierre Kinet (Beth Israel Deaconess Medical Centre, Boston, Massachusetts, USA). The NFAT-DsRed and RS-ATL8 cells were produced in the authors’ laboratories in Nottingham (FHF, MJCA) and Tokyo (RN), respectively, and are also available through an MTA.

All RBL cell lines were cultured in Minimum Essential Medium supplemented with 10% v/v heat-inactivated foetal bovine serum, 100 U/mL penicillin, 100 μg/mL streptomycin and 2mM L-glutamine (RBL medium). Depending on the transfected cell line, the RBL medium contained one or two additional antibiotics, as follows: RBL 703/21, 1 mg/mL G418 sulphate (Thermo Fisher Scientific, UK); RBL SX-38, 1 mg/mL G418 sulphate; NFAT-DsRed, 20 μg/mL blasticidin S (Invivogen, USA) and 1 mg/mL G418. RS-ATL8, 200 μg/mL hygromycin B (Invitrogen, UK) and 500 μg/mL G418 sulphate. Details of the subculturing were as described by Wan *et al*. [15]. Routine mycoplasma testing was carried out using a PCR-based Mycoplasma Test Kit II (PromoKine, Germany). Ethical approval for the use of human atopic serum was given by the University of Nottingham School of Pharmacy Research Ethics Committee (Ref 047–2018).

### RT-PCR

Total RNA isolation and on-column cDNA synthesis was carried out using Miltenyi Biotec’s μMACS One-step cDNA Synthesis Kit, following the manufacturer’s recommended protocol using oligo-dT for priming. The relevant genes were amplified by PCR using following species-specific (subscript H = human, R = rat) oligonucleotide primers (Sigma-Aldrich, UK), with the expected amplicon sizes given in brackets for cDNA and genomic DNA (Table 1). Species-specificity was checked using Primer BLAST [16] and optimal annealing temperature validated experimentally using temperature gradients.

**Table 1:** Sequences and predicted amplicon sizes for oligonucleotides primers used in this study.

Transcript target	Primer direction	Oligonucleotide sequence (5’→3’)	cDNA / gDNA product size (bp)
βACT_R_	Forward	TGAGAGGGAAATCGTGCGTG	278 / 368
	Reverse	TGTTGGCATAGAGGTCTTTACGG	
GAP-DH_H_	Forward	TGATGACATCAAGAAGGTGGTGAAG	240 / 240
	Reverse	TCCTTGGAGGCCATGTGGGCCAT	
FcεRIα_H_	Forward	AATGGCAGCCTTTCAGAAGA	360 / 2165
	Reverse	CTCATAGTCCAGCTGCCACA	
FcεRIβ_H_	Forward	TCCTGGACAGCTCGGTTAAT	338 / 1722
	Reverse	TCCCCAGAATGGATAACCTG	
FcεRIγ_H_	Forward	GGAGAGCCTCAGCTCTGCTA	218 / 946
	Reverse	CATCTATTCTAAAGCTACTGTGGTGGT	

The PCR step was run on a PTC-200 Peltier thermal cycler (MJ Research, USA) using the following cycling conditions: 2 min initial denaturation, followed by 35 cycles of denaturing (30 sec, 94°C), annealing 45 sec, 60°C) and extension (90 sec, 72°C) followed by a final extension (5 min, 72°C). Each 20 μL polymerase chain reaction (PCR) for analysis was made up by mixing 10 μL 2x GoTaq Hot Start Green Master Mix (Promega, UK), 7 μL molecular biology-grade water, 1 μL 10 μM (500 nM final concentration) of each appropriate forward and reverse oligonucleotide primer and 1μL DNA template. PCR product sizes were verified on agarose gel electrophoresis (1% w/v) in 0.5x Tris/borate/EDTA buffer (ThermoScientific Fisher, UK) alongside 100 bp TriDye DNA ladders (New England Biolabs, USA) before imaging under UV light using a GeneGenius Gel Imaging System (Syngene, UK) using a 400ms exposure for image capture.

For quantification of differences in FcεRIα mRNA levels, cDNA prepared as above was amplified using SsoAdvanced Universal SYBR Green Supermix (BioRad, UK) and the pre-designed PrimePCR oligonucleotide primers from BioRad for rat GGT1 (reference gene) and human FCER1A (target gene) described below in section “Gene Copy Number determination by qPCR” using the same cycling conditions, but were run on an Mx3005P QPCR System (Agilent, UK) and analysed using MxPro QPCR software (Agilent, UK) and the Pfaffl equation (19).

### Amplification and sequencing of FCER1A cDNA

500 ng of cDNA of each of the four cell lines, obtained as described in the previous section, were amplified by PCR using a mixture of GoTaq Hot Start Green Master Mix (Promega, UK) and Q5 DNA proofreading polymerase (New England Biolabs; 1 μL added per 20 μL volume) and nuclease-free water, with the following primers: 5’-ACAGTAAGCACCAGGAGTCC-3’ and 5’- ATATTGCAAGCTGTGTTTGACA-3’. The primers bind to the 5’ and 3’ UTRs of the human FCER1A gene, respectively, amplifying a 889 bp product, and therefore do not modify the sequence in the coding region of the FCER1A cDNA. Cycling was carried out using MJ Research PTC-200 Peltier Thermal Cycler, using the following parameters: 5 min initial denaturation at 94°C, 35 cycles of 30 seconds at 94°C, 45 seconds at 56°C and 1 min at 72°C, followed by a final elongation of 10 min. The reaction was then cooled at 5°C until stopped. PCR products were gel- extracted using Qiagen Gel Extraction kit as directed by the manufacturer and sequenced using the following primers: FCER1A FOR: 5’-GCCATGGAATCCCCTACTCT-3’; FCER1A REV: 5’-TGTTTTTGGGGTTTGGCTTA-3 FCER1A_int FOR: 5’- TTACAAATGCCACAGTTGAAG-3’, FCER1A_int REV: 5’- ACCAGTACTTGAGAGCTTCAC-3. Sequencing results were analysed using SnapGene and compared with the GenBank reference sequence with the Accession number NM_002001.3. All oligonucleotide primers were manufactured by Merck. Specificity of the primers was ensured by blasting the sequences against the human and rat genomes using Primer-BLAST (16). All sequencing was performed by Source BioScience (Nottingham, UK).

### Gene copy number determination by qPCR

For assessment of transgene copy number using qPCR, genomic DNA was extracted from RBL-2H3 (negative control), RBL-703/21, RBL-SX-38, RS-ATL8 and RBL-NFAT-DsRed using DNeasy Blood and Tissue Kit (QIAGEN, UK) as directed by the manufacturer. Genomic DNA was used for quantification of FcεRIα_H_ in comparison with Rat gamma-glutamyltransferase 1 (GGT1_R_). This gene was chosen based on its description as a single copy gene [17] in the rat genome. The following pre-designed PrimePCR primers from BioRad were used: Rat gamma-glutamyltransferase 1 (CD224) Ggt1 primer (qRnoCED0003031; exonic; amplicon length 116 bp; efficiency: 97%), and human high affinity IgE receptor alpha chain (FCER1A) (qHsaCID0005954; Intron-spanning; amplicon length 102 bp; efficiency 98%).

qPCR reactions were performed in a 20 μL volume containing 10 μL of SsoAdvanced Universal SYBR Green Supermix (BioRad), 1 μL of the pre-designed FcεRI_H_ primer pair in one reaction and Ggt1_R_ primer pair in the second reaction, 1 μL of genomic DNA template (final concentration 100 ng/μL) and 18 μL of molecular biological grade water. PCR reaction was performed using CFX96 Real time system with C1000 Touch Thermal Cycler (BioRad). Cycling conditions were as follows: 95°C for 2 min, (95°C for 5 seconds, and 60°C for 30 seconds) repeated 39 times, 95°C for 5 seconds, 65°C for 5 seconds and 95°C for 5 min, followed by a melt curve.

Relative quantification of transcripts was performed as described by Sommeregger *et al*. [18] using the equation described by Pfaffl [19], which takes into account the efficiencies of the primers.

### Receptor quantification assay

Quantum Simply Cellular Microsphere vials (QSC, Bangs Laboratories, Polysciences) were shaken well for uniform suspension. One drop of QSC microspheres was added to 50 μL of DPBS buffer. Then, microspheres were stained (except the blank) by the addition of 10 μL of the labelled APC anti-human FcεRIα antibody (BioLegend, AER-37 (CRA-1)) and incubated on ice in the dark for 30 min. Next, microspheres were washed twice by adding 1 mL of DPBS buffer and centrifuged at 2500 x g for 5 min. Finally, the microspheres were re-suspended in 500 μL of DPBS buffer and transferred to FACS tubes, and each population stained separately. 2X10^6^ cells from each cell line (RBL-2H3, RBL-703/21, RBL-SX-38, RS-ATL8 and RBL-NFAT DsRed) were cultured in a 48-well plate (Nunc UpCell, Thermo Scientific Fisher) after overnight sensitization of the cells with 1 μg/mL human IgE (BioPorto, Hellerup, Denmark). These cell culture dishes are coated with a temperature-responsive polymer, which allows cell attachment at 37°C and cell harvesting by decreasing the temperature, without the need for enzymatic detachment reagents. This avoids potential issues with downstream surface receptor expression analysis by flow cytometry due to trypsin degradation.

The next day, cells were harvested by letting the plates stand at room temperature for 30 min and transferred to a FACS tube. Cells were then centrifuged at 600 x g for 5 min and the supernatant discarded. An optimal saturating amount of 5 μL of the labelled APC anti-human FcεRIα antibody (BioLegend, Clone AER-37 (CRA-1)), which had been previously determined, was added to the cell suspension and incubated for 30 min on ice in the dark. Cells were then washed twice with 4 mL of DPBS buffer (Sigma Aldrich, UK) and centrifuged at 600 x g for 5 min. After the second wash, cells were resuspended in 500 μL DPBS and analysed using a Beckman Coulter FC500 flow cytometer. Microspheres and cells were analysed with the same settings to ensure accurate and reproducible assignments.

All flow cytometry experiments included parental RBL-2H3 cells as negative control, which except for the introduced human genes will have the same endogenous receptors as the humanized derivatives. Therefore, we did not use any isotype controls.

### Transfection with human FcεRI gamma chain

pBJ1 neo-hu FcεRI gamma was a gift from Jean-Pierre Kinet (Addgene plasmid #16540) [20] and was propagated in *E*. *coli* using standard molecular biology techniques. Before nucleofection, cells were checked under the light microscope to be 80% confluent, since the optimal confluency for nucleofection is 75–80%. For each nucleofection, 2 μg FcεRI gamma plasmid was transfected in 4x10^6^ cells in 100 μL cell culture medium in Nucleocuvette vessels using the SF Cell Line Optimization 4D-Nucleofector XL kit (Lonza, UK). To determine transfection efficiency, an equal amount of cells were transfected using 2 μg pmaxGFP vector, constitutively encoding a green fluorescent protein. Post nucleofection, 700 μL transfected cells resuspended in fresh warm medium were transferred into a clear, flat-bottomed, tissue-cultured treated 6-well polystyrene plate (Corning, UK) containing 1 mL fresh warm medium without antibiotics and placed in a 37°C cell incubator for 24h. Transfected cell images were then taken using an Evos *fl* Digital Inverted Microscope. For green fluorescence, the GFP light cube was used (470 nm excitation, 525 nm emission). Images were taken at 10x magnification, and transfection efficiency calculated as the percentage of green fluorescent cells in the total cell population. 24h post transfection, fresh warm medium was added to the transfected cells; this was supplemented with 600 μg/mL Hygromycin B (Invivogen, UK), based on kill curves determined in preliminary experiments. Cells were kept under the selective antibiotic pressure for three weeks. Next, surviving transfected cells were transferred to a 24-well Upcell cell culture dish containing 2x10^6^ cells in each well in a total volume of 500 μL and processed for flow cytometry or RT-PCR.

### Staining for flow cytometry

To block endogenous Immunoglobulin receptors, cells were incubated with 2% rat serum (Sigma-Aldrich, UK) for 4h at 37°C. Cells were then washed once with Phosphate buffered saline (PBS) (Merck, UK), and sensitized with 1 μg/mL human IgE (BioPorto) for 16 h at 37°C. After this incubation, cells were kept at room temperature for 30 min in order to detach them from the plate surface and then transferred to labelled FACS tubes. Subsequently, sensitized cells were stained with 5 μl FITC- or APC-labelled anti-human FcεRIα antibody (BioLegend, AER-37 (CRA-1)) and washed three times with 4 mL DPBS until performing FACS analysis using a Beckman Coulter FC500 Cytometer.

## Results

**Fig 1:**
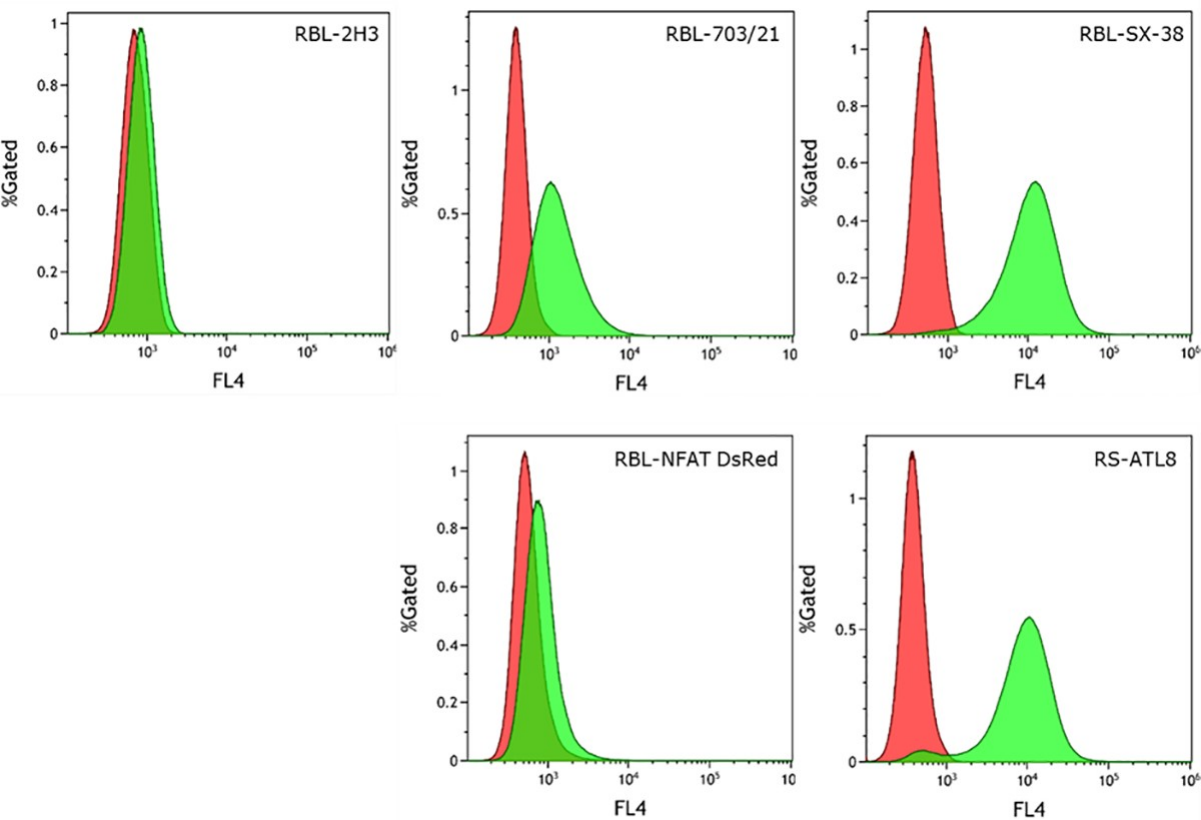
Surface expression of FcεRIα_H_ receptor on humanized RBL cells determined by flow cytometry after staining with APC-labelled mouse-anti-human FcεRIα antibody (clone AER-37/CRA-1). Red histograms: unstained cells, green histograms: stained cells. Cells were sensitized with 1 μg/mL human IgE (BioPorto) for 16 h prior to labelling. Representative of five biological replicates.

We first quantified the expression levels of FcεRIα_H_ in SX-38, RS-ATL8, RBL-703/21 and NFAT-DsRed cell lines using flow cytometry, using the parental RBL-2H3 cell line as a negative control. Staining with the monoclonal CRA-1 antibody specific for human FcεRIα_H_-chain (Fig 1) showed that while the RS-ATL8 and its parental line SX-38 had very similar, high FcεRIα_H_ expression, the NFAT-DsRed and its parental RBL 703/21 line had much lower expression.

**Fig 2:**
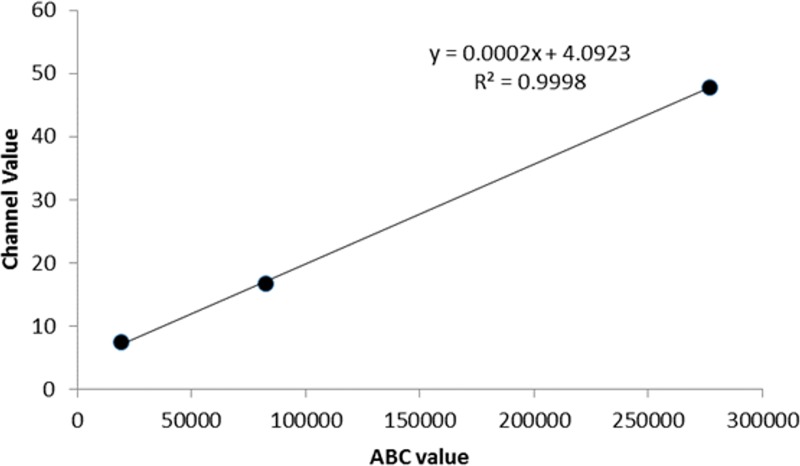
Microsphere correlation curve. Calibration beads were stained with APC anti-human FcεRIα antibody and analysed by flow cytometry to obtain the channel value. The affinity binding capacity (ABC value), proportional to the number of receptors on the cell surface, is given next to the calibration curve (n = 1).

Using calibration microspheres, and the same CRA-1 anti-FcεRIα_H_ APC-labelled antibody, we found SX-38 and RS-ATL8 cells to have approximately 450,000–500,000 FcεRIα_H_ molecules per cell (Fig 2 and Table 2), which is similar to the maximal number of receptors described on human peripheral blood basophils (up to 600,000 /cell)[21], while RBL 703/21 and NFAT-DsRed cells appeared to have ~52,000 and ~16,000 human α-chains per cell, respectively.

**Table 2:** Extrapolated Affinity binding capacity (ABC), proportional to FcεRIα_H_ surface receptors per cell.

Cell Line	ABC value
SX-38	453,038
RS-ATL8	507,038
RBL 703/21	52,538
NFAT-DsRed	16,538

**Fig 3:**
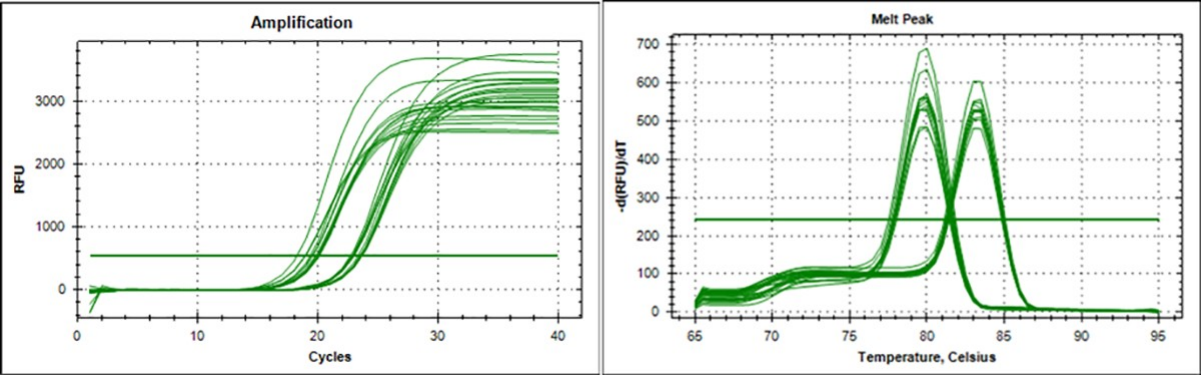
Amplification of FcεRIα_H_ and GGT1 in RBL-SX-38, RBL-703/21, RS-ATL8 and NFAT DsRed A: amplification curve of humanized RBL cells. B: Melt peak analysis for FcεRIα_H_ and GGT1 amplicons obtained by PCR with genomic DNA of the humanized RBL-cells. RBL-2H3 genomic DNA does not result in amplification of a product with FcεRIα_H_-specific primers (S1 Fig).

Next, we aimed to determine whether the differences in surface expression of the transgenic FcεRIαH chain could be due to differences in gene copy numbers. Integration of transfected cDNA in stable transfectants is a random recombinational event [22], giving rise to a polyclonal population of stably transfected cells with varying gene copy numbers integrated into their chromosomal DNA. In order to determine how many copies of FcεRIαH cDNA had been inserted into the RBL genome, we amplified FcεRIα_H_ and Ggt1_R_ (as a single copy reference gene) by qPCR, expressing the data as a relative expression ratio of FcεRIα_H_/Ggt1_R_. Results for four independent experiments, performed in triplicates, are shown in Table 3. Fig 3 shows corresponding qPCR amplification curves and melt point analysis confirming that a single amplicon was amplified by the primers in both cases.

**Table 3:** Assessment of FcεRIα_H_ gene copy number, expressed as a relative expression ratio of FcεRIα_H_/Ggt1_R_. Data are from 4 separate independent experiments, each performed in triplicates.

	RBL-SX-38	RBL-703/21	RS-ATL-8	RBL-NFAT-DsRed
Expt. 1	9.3 : 1	30.3 : 1	8.2 : 1	9.4 : 1
Expt. 2	9 : 1	26.4 : 1	7.7 : 1	9 : 1
Expt. 3	9.3 : 1	25 : 1	8.6 : 1	10.3 : 1
Expt. 4	9 : 1	32 : 1	8.6 : 1	10 : 1
Rel. ratio FcεRIα_H_/Ggt1_R_ Mean±SD	9.15:1 ± 0.15	28.42:1 ± 2.8	8.28:1 ± 0.37	9.68:1± 0.44
Range	9	25–32	8–9	9–10

All humanized cell lines appeared to possess approximately 8–9 copies of FcεRIα_H_, with the exception of the RBL 703/21 cell line, which had a higher number of transgene copies (~28) and the highest variation between experiments (25–32). This could suggest that the RBL 703/21 is not (any more) a clonal population, in contrast to the other three cell lines, which have undergone a documented clonal selection. This however is not reflected in the flow cytometry data (Fig 1), which show a single peak for RBL 703/21, whereas the presence of an unstained cell population in the RS-ATL8 cells could also suggest some degree of heterogeneity.

**Fig 4:**
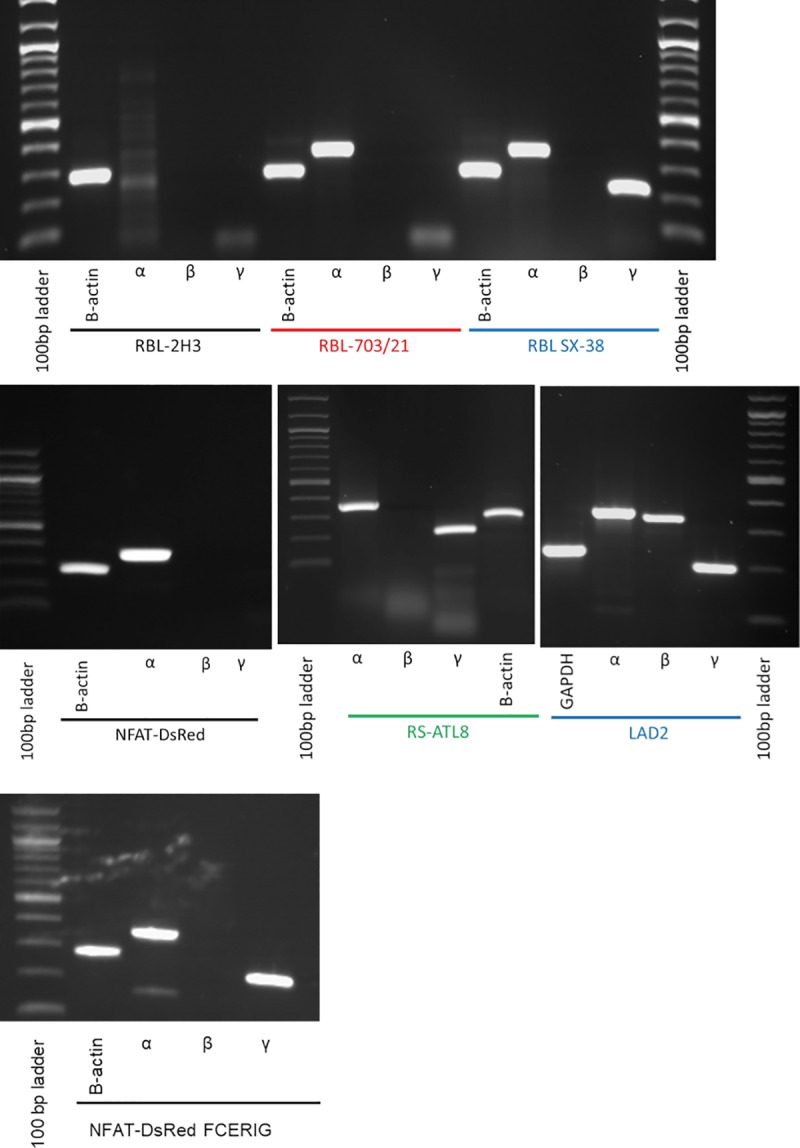
RT-PCR demonstrating differential expression of the human FcεRIα, FcεRIβ and FcεRIγ chains in non-humanized parental RBL-2H3 cell line (no expression), RBL 703–21 and NFAT-DsRed (FcεRIα_H_ only), SX-38 and RS-ATL8 (FcεRIα_H_ and FcεRIγ_H_ only) and LAD-2 (FcεRIα_H_, FcεRIβ_H_ and FcεRIγ_H_ chains). The stably transfected NFAT-DsRed FCERIG also showed FcεRIα_H_ and FcεRIγ_H_ expression. Representative of three independent biological replicates with comparable results.

Our next step was to assess and compare the mRNA expression of the human FcεRIα, FcεRIβ and FcεRIγ chains in the four humanized cell lines by RT-PCR. Messenger RNA was isolated and reverse transcribed into single stranded cDNA, which was subjected to PCR using primers specific for the human subchains of the FcεRI receptor. Housekeeping genes (rat β-actin for RBL, human GAP-DH for the human mast cell line LAD-2) were included as positive controls. As shown in Fig 4, the primers specific for the three human FcεRI subchains did not amplify any major discrete band from non-humanized RBL-2H3 cells, confirming that the primers are specific for the human genes. The primers for human FcεRIα did generate a weak, non-specific pattern of amplicons with the RBL-2H3 cell line that can be clearly discriminated from the single band obtained with the human mast cell line LAD-2, which was used as positive control for all three human subchains. While both RBL 703/21 and NFAT-DsRed only expressed human FcεRIα, the RBL-SX-38 and RS-ATL8 expressed human FcεRIα and FcεRIγ, but not the FcεRIβ chain. This result is consistent with the known genesis of the reporter cell lines from their humanized precursors (NFAT-DsRed from RBL703/21 and RS-ATL8 from RBL SX-38).

In order to rule out that differences in expression levels were due to sequence differences in the FcεRIα_H_ cDNA (known endoplasmatic reticulum retention signals will be discussed in detail in the discussion section below), the complete FcεRIα_H_ sequence was amplified by PCR from cDNA and the PCR product directly sequenced. The results demonstrated in all four humanized cell lines a 100% identity with full coverage of the reference FCERIA sequence available in GenBank (Accession Number NM_002001.3) (see S2 Fig).

Because the integration of transgenes into chromosomal DNA is a random event, it is possible that the differences in protein surface expression levels might be dictated by differences in steady state transcriptional levels of FcεRIα_H_ mRNA. To ascertain whether this is the case, we measured mRNA levels in all four humanized cell lines by RTqPCR, using rat GGT1 as reference gene for normalization. Relative expression levels, compared with the FcεRIα_H_ mRNA levels in SX-38, are summarised in Table 4.

**Table 4:** Results of RT-qPCR experiments showing levels of FcεRIα_H_ mRNA expression relative to SX-38 cells. cDNA from all 4 cell lines was amplified by qPCR using SybrGreen and mRNA levels determined using rat GGT1 as reference gene. Results are from 3 biological replicates, each performed in triplicate determination.

	Expt. 1	Expt. 2	Expt. 3	mean ± st. dev.
RBL 703/21	3.5	4.8	3.3	3.87 ±0.81
NFAT-DsRed	1.6	2.2	2	1.93 ±0.31
RS-ATL8	1.36	0.75	1.18	1.10 ±0.31
SX-38	1	1	1	1.00

The mRNA levels for the α-chain show ~3.9-fold higher expression levels for RBL 703/21 cells, which correlates well with the ~3.5-fold higher gene copy number in this cell line compared with the other three, which all had comparable levels. NFAT-DsRed still appeared to have ~1.9-fold elevated levels of α-chain mRNA compared with SX-38, while the RS-ATL8 levels were very close to those in the parental cell line SX-38.

Next, we asked whether transfection of NFAT-DsRed reporter, which showed the lowest FcεRIα_H_ surface expression, could be increased by transfection with FcεRIγ_H_. This assumption was based on the observation that both humanized cell lines which co-expressed FcεRIα_H_ and FcεRIγ_H_ (RS-ATL8 and its parental SX-38) had the highest FcεRIα_H_ surface expression, while the expression of this receptor was much lower in the single α-chain transfectants.

**Fig 5:**
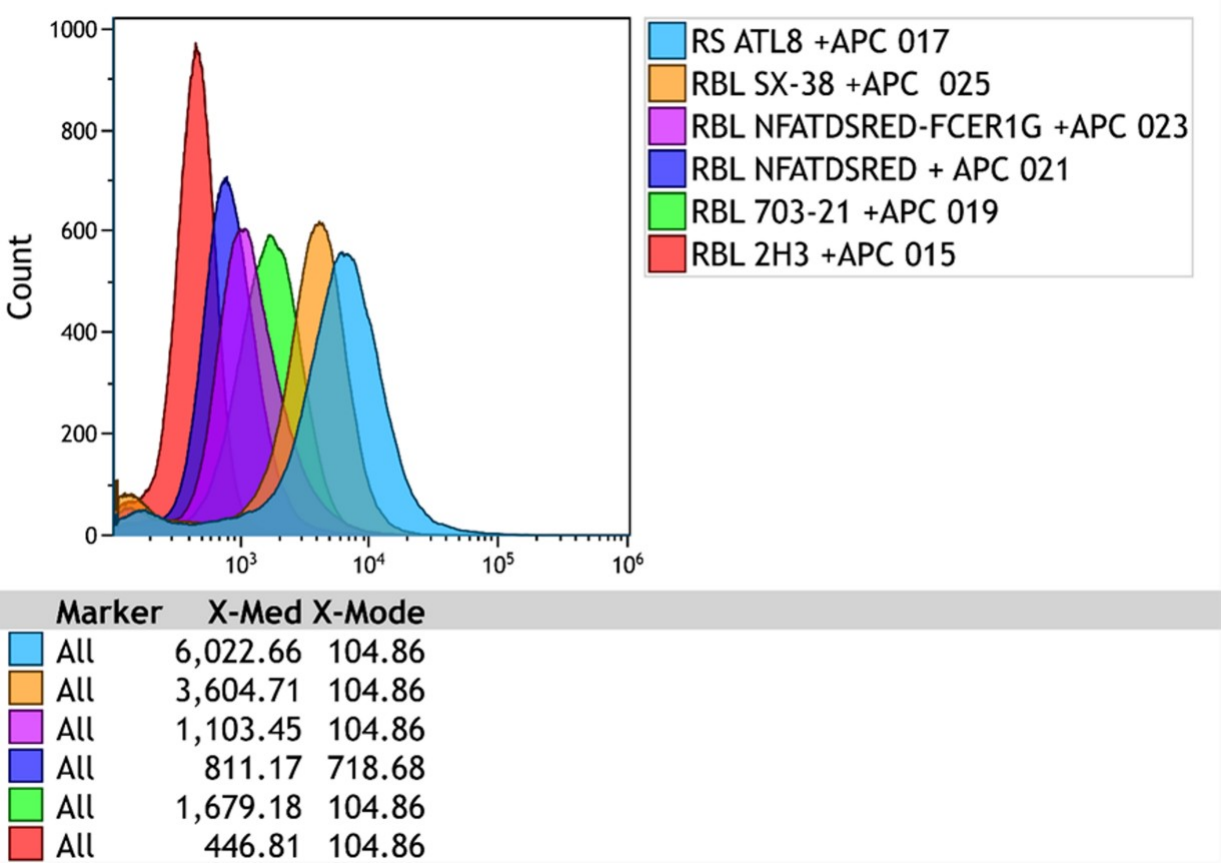
Histograms of NFAT DsRed cells stably transfected with pBJ1 neo-hu FcεRI gamma (NFAT DsRed FCERIG), encoding cDNA for the human FcεRI gamma chain under the control of a constitutive promoter. The blue histogram shows untransfected NFAT-DsRed cells, the stably transfected NFAT-DsRed cells in comparison with the RS-ATL8 cells. All cells were stained with FITC-labelled anti-human FcεRI α-chain monoclonal antibody (CRA-1). Result is representative of 4 independent biological replicates.

As shown in Fig 5, NFAT-DsRed stably transfected with a plasmid encoding the FCER1G cDNA with a constitutive promoter resulted in almost doubling of the median fluorescence compared with the non-transfected cells. This result demonstrates how co-expression of FcεRIα_H_ and FcεRIγ_H_ chain in RBL can restore higher levels of surface FcεRIα_H_ expression, albeit still not to a level comparable with SX-38 and RS-ATL8. This may suggest the existence of additional factors governing FcεRI cell surface expression.

**Fig 6:**
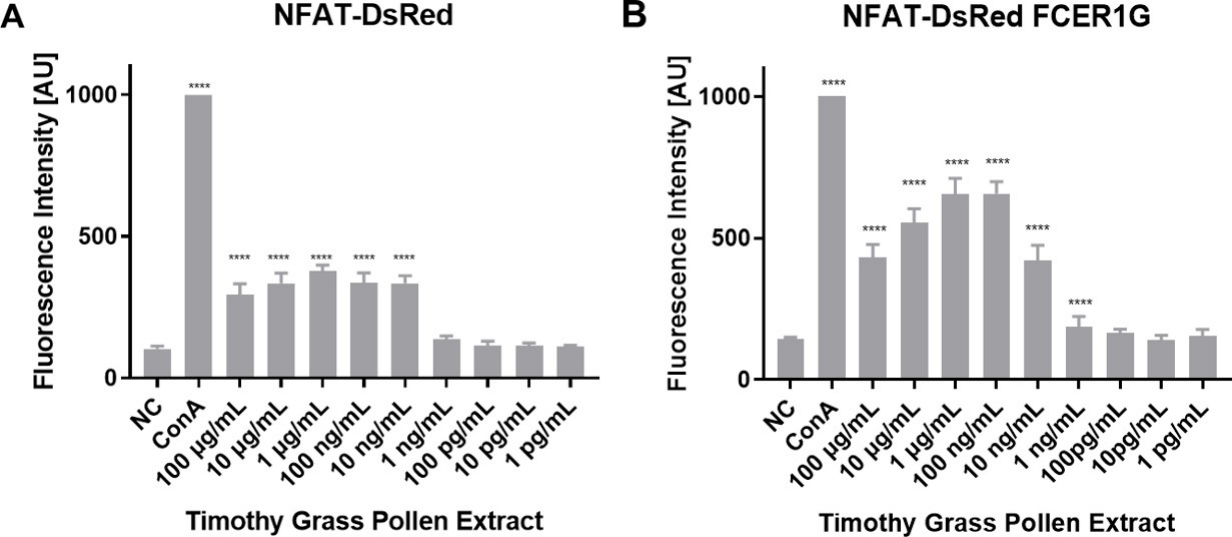
Dose response curve showing the increased response of the FCER1G-transfected NFAT-DsRed reporter. Both cell lines were sensitized overnight with serum of a grass-pollen allergic individual (diluted 1:50) and stimulated with a serial dilution of Timothy grass pollen extract the next day. Fluorescence was measured after 16–18 hours of incubation. Data were normalized for ConA (1 μg/mL), used as positive control. Representative of 4 biological replicates, each performed in triplicates.

Our next step was to assess whether the increased FcεRIα_H_ surface levels in the NFAT-DsRed human FCER1A/FCER1G double transfectant cells resulted in a higher response than the single human FCER1A only transfectant parental cell line. The results in Fig 6 show that an enhancement of sensitivity has been obatined by stable transfection of the FCER1G cDNA. The fluorescence measured after activation under identical conditions is higher and the detection limit is increased 10-fold from 10 ng/mL allergen extract in the single transfectant to 1 ng/mL in the double transfectant.

## Discussion

High affinity IgE receptor expression on peripheral blood basophils has been shown to vary largely (>10 fold) between donors, and even between different assessments of the same donor [23]. The situation will be different in the humanized reporter cell lines, which do not have the same variation, as they are derived from the same parental cell lines and are of clonal origin. The surface expression of FcεRI is regulated by several mechanisms operating at different levels. As FcεRI levels have a direct impact on the ability of the IgE reporter systems to be sensitized by human IgE, determining the overall reporter system’s sensitivity, the operating regulatory mechanisms need to be briefly summarized here.

First, all four humanized RBL cell lines are all stably transfected with the human FcεRIα chain under the control of strong synthetic constitutive promoters. Therefore, mechanisms underlying transcriptional regulation of human FcεRI [24] in these transfectants are irrelevant and will not be discussed here. The relevant regulatory mechanisms all operate at the post-translational level. A key requirement for successful export of the FcεRI α-chain has been described as the N-linked glycosylation in the endoplasmic reticulum (ER), in particular the removal of terminal glucose residues by glucosidases [25]. Furthermore, it has been shown that the presence of the FcεRI γ-chain is necessary as in the absence of the γ-chain, the α-chain folds correctly, but accumulates in the ER. Letourneur *et al*. [26] demonstrated that the two lysine residues located in position -3 and -7 of the PKPNPKNN C-term of the α-chain (Lys^226^ and Lys^230^) act as ER retention signals, but also highlighted the existence of other retention signals elsewhere. Dilysine residues at positions -3 and -4 near the cytosolic end are a commonly found ER retention signal in eukaryotic cells [27]. The authors specify that these are not true retention signals, instead they act as retrograde transport signal from the Golgi compartment back to the ER. In basophils and mast cells, the α-chain associates with two γ-chains, masking the retrograde transport signal, hence leading to loss of ER retention. An additional dilysine sequence (Lys^212^-Lys^216^), located closer to the single transmembrane domain of the α-chain, has also been shown to play a role in regulating surface expression, as does an unusual charged residue (Asp^192^) inside the transmembrane domain [28]. These signals are possibly the additional signals postulated by Letourneur and colleagues [26]. We have sequenced the human α-chain cDNA used for transfection of the humanized reporter systems RBL-703/21, NFAT-DsRed, SX-38 and RS-ATL8 and found them to be identical (see S1 Fig). All cDNAs had the five retention signals (D192, K212, K216, K226 and K230) described above, and no other differences were found, neither at amino acid nor at the DNA level.

The SX-38 humanized RBL cell line and the RS-ATL8 luciferase reporter derived from it are generally considered triple human FcεRI αβγ transfectants. However, our RT-PCR data point to the loss of the transfected β-chain in the parental SX-38 cell line, and consistently also in the RS-ATL8 line derived from it. The loss of the human β-chain could be due to the lack of selection markers on the pCDL-Srα296 plasmid which was used for transfection of RBL-2H3 with the human α- and β- chains [5][29]; in contrast, the human γ-chain was introduced using pBJ1neo, which contains a Neomycin resistance gene for selection in mammalian cells [5].

The humanized RBL703/21 and NFAT-DsRed, which was derived from the former, are single α-chain transfectants. This fact might explain why the surface levels of the human FcεRI α-chain are lower in these two humanized RBL cell lines than the SX-38 and RS-ATL8. Taudou *et al*. [30] suggested that the human FcεRI α-chain can associate with the rat FcεRI y-chain, but this interaction is less efficient in masking the retention signals. This suggests that in the absence of an interaction with the human γ-chain in the ER, the surface expression of the α-chain in the single transfectant RBL-703/21 and NFAT-DsRed will be reduced, which our data confirms. This would also explain why introducing the human FcεRIy chain by transient transfection, as shown in Fig 6, was able to increase (albeit, unexpectedly, to a limited extent) human FcεRIα surface expression. It is possible that the lower-than-expected human alpha chain (FcεRIα_H_) surface expression is due to different levels of expression of the mRNA encoding the human gamma chains (FcεRIγ_H_) between the different humanised cell lines, resulting in different levels of competition between the endogenous rat and the introduced human gamma chains at the protein level for assembly into the tetrameric receptor complex.

Finally, in terms of posttranslational regulation of FcεRI expression, work by Platzer and co-workers [31] suggested that the signal peptide of the FcεRI α-chain itself plays a role in regulating surface expression. Swapping the natural signal peptide for that of H2-K^b^ significantly increased surface expression of the α-chain, with or even without the γ-chain. Once the FcεRI receptor has reached the surface, the binding of exogenous IgE stabilizes it [32]. All transfected cell lines contained the original, natural signal peptide, so this cannot account for differences in expression levels.

The observation that, in the absence of any sequence differences in the coding region of the α-chain between the four cell lines (S2 Fig) or differences in steady state levels of α-chain mRNA (Table 4), stable transfection with the γ-chain only partially restores surface levels, suggests that other, hitherto unknown regulatory mechanisms might be operating. An additional requirement for the simultaneous presence of the human β-chain appears unlikely, as this is also not present in the SX-38 and RS-ATL8 higher FcεRIα_H_ expressing cell lines.

As the ability of allergens to successfully induce basophil/mast cell activation hinges critically on the amount of allergen-specific IgE present on the cell surface, FcεRI α-chain surface expression levels are a key parameter. Despite the 30 times lower surface expression of FcεRIα_H_-chain, the NFAT-DsRed responds well to IgE-dependent stimulation, as it was derived by two rounds of FACS sorting followed by cloning, resulting in a highly reactive clone. The twice-sorted clone has a 8-fold higher sensitivity than the single sorted and a signal to noise ratio of ~30fold (S3 Fig), in line with the RS-ATL8 luciferase reporter. However, while both the luciferase (RS-ATL8) and red fluorescent (NFAT-DsRed) reporter cell lines work equally well with high to intermediate concentrations of allergen-specific IgE, as a result of the ~50-fold lower surface expression of FcεRIα_H_, the NFAT-DsRed fails to yield a robust signal with lower IgE concentrations. In contrast, the RS-ATL8 in our hands can detect as little as 100 pg/mL IgE when stimulated polyclonally with an anti-IgE antibody; it will also respond positively to 1 pg/mL of allergen after sensitization with serum of individuals with a matching allergy [11][13].

In the presence of low IgE serum concentrations and low surface expression of FcεRIα_H_-chain, the RBL assay can result in a false negative result due to insufficient sensitization of the reporter cell line. We have suggested use of the humanized RBL reporters for assessment of potential allergenicity of vaccine candidate antigens [13]. In particular in the context of anti-helminth vaccination of individuals living in helminth-endemic areas, who have potentially high levels of parasite-specific IgE in their blood and on their basophils and mast cells, a low FcεRIα_H_ surface expression in the reporter systems used for screening may represent a hazard, as mast cells in the skin and tissues of vaccinees would have a significantly higher number of FcεRI receptors on their surface, thus a lower activation threshold. This could result in a failure of such safety assessment assays to identify potential allergenicity, and ultimately result in a systemic urticarial reaction scenario, such as the one encountered during a clinical trial for vaccination with the hookworm candidate Na-ASP-2 [33]. Therefore, if humanized reporter systems are to be used for vaccine safety (allergenicity) assessment, their FcεRIα_H_ expression levels should be at least comparable with those in human cells.

Despite the lower FcεRIα_H_ expression levels, and its shortcomings with low IgE concentrations, the NFAT-DsRed has one distinct advantage over its RS-ATL8 luciferase sibling, as it does not need any expensive luciferase reagents, and can be used for screening allergenicity using allergen microarrays. This enables the simultaneous screening of multiple (i.e. several hundreds) of allergens in array (or multiwell format) at a lower cost. Therefore, our future efforts will be directed at obtaining a fluorescent humanized reporter assays with FcεRIα_H_ expression levels comparable to the RS-ATL8.

## Supporting information

S1 Fig **Example of PCR amplification of human FcεRIα (A) and GGT1 gene (B)** from parental RBL-2H3 cells, from which all humanized cell lines described in this paper were derived. Genomic DNA was extracted from RBL-2H3 cells and subject to PCR amplification as described in Materials and Methods using the gene of interest (HsFcεRI) primers (A) and the reference gene (rat GG1) primers (B). As expected, there is no amplification with the human FcεRIα-specific primers, demonstrating lack of amplification of the endogenous homologous FcεRI rat gene.
(PDF)

S2 Fig A) Map (SnapGene) of the coverage obtained from sequencing the cDNA with 6 different primers. The binding positions and names of the primers are indicated at the top of the map in purple. The bottom of the map also indicates the positions of the signal sequence peptide, the transmembrane domain and the 5 ER retention signals (RS) D192, K212, K216, K226 and K230. Full multiple coverage was obtained for all four humanized RBL cell lines, demonstrating complete identity of the cDNA sequences. B) Details of the chromatograms in the region containing the five known retention signals.
(PDF)

S3 Fig A polyclonal population of stably transfected NFAT-DsRed cells was sensitized overnight with 1 μg/mL IgE and activated with 2 μg/mL polyclonal goat anti-human IgE the next day. After a further incubation of 16–18 hours, responding cells producing DsRed were sorted by flow cytometry as single cells into 96-well plates, and clones allowed to grow and expand for several weeks. The highest responding cells were pooled and the process, consisting of activation, sorting and cloning was repeated once more. Individual 2x sorted clones were expanded and tested for their response to anti-IgE. A) shows the response of the 1x sorted cells, a 2x sorted high responding clone (A6) and an intermediate responding clone (H8) to activation via the IgE receptor (2 μg/mL anti-IgE) or 1μg/mL A23187. After removal of the medium, cells were lysed in 1% v/v Triton X-100 in DPBS and the lysate transferred to low-autofluorescence black plates. Fluorescence was read in an Infinite M200 plate reader (Tecan, Männedorf, Switzerland), using 530nm excitation and 590nm emission filters (this gave better results than the reported optimal 554nm excitation and 591nm emission for DsRed-Express2). B) shows the same cells with and without IgE-dependent activation under the EVOS *fl* microscope at 100x magnification using the RFP light cube.
(PDF)
