# Supplementary figures and images for: Characterization of human FcεRIα chain expression and gene copy number in humanized rat basophilic leukaemia (RBL) reporter cell lines
Source: PLoS One. 2019 Aug 20;14(8):e0221034. doi: 10.1371/journal.pone.0221034 (PMC6701790; doi:10.1371/journal.pone.0221034)

S1 Fig

A

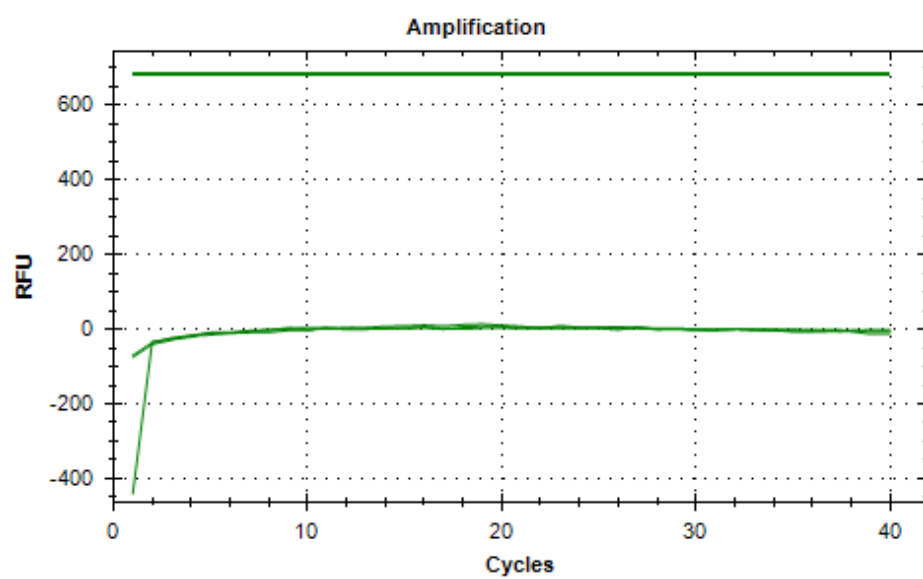

B

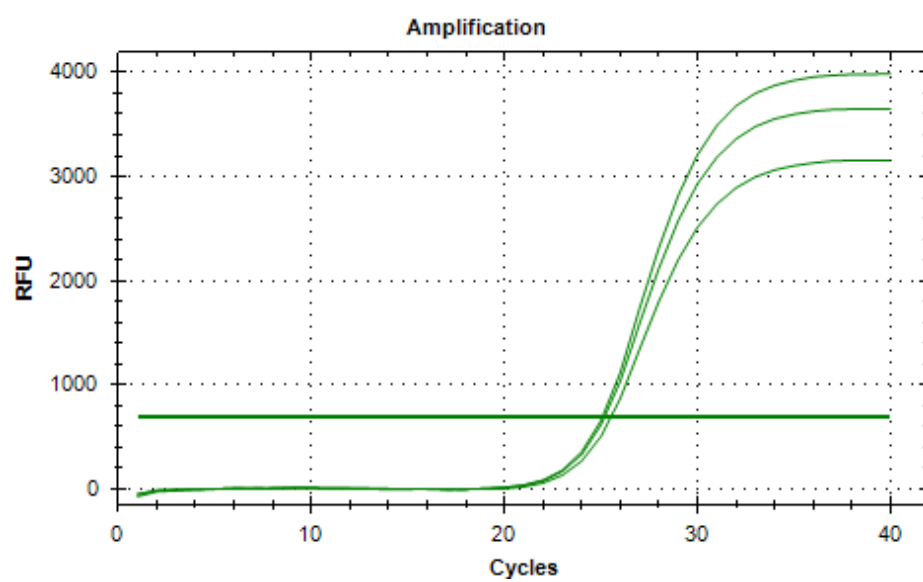

Supplement: S1 Fig — Example of PCR amplification of human FcεRIα (A) and GGT1 gene (B) from parental RBL-2H3 cells, from which all humanized cell lines described in this paper were derived. Genomic DNA was extracted from RBL-2H3 cells and subject to PCR amplification as described in Materials and Methods using the gene of interest (HsFcεRI) primers (A) and the reference gene (rat GG1) primers (B). As expected, there is no amplification with the human FcεRIα-specific primers, demonstrating lack of amplification of the endogenous homologous FcεRI rat gene. (PDF) [file pone.0221034.s001.pdf]

S2 Fig

A

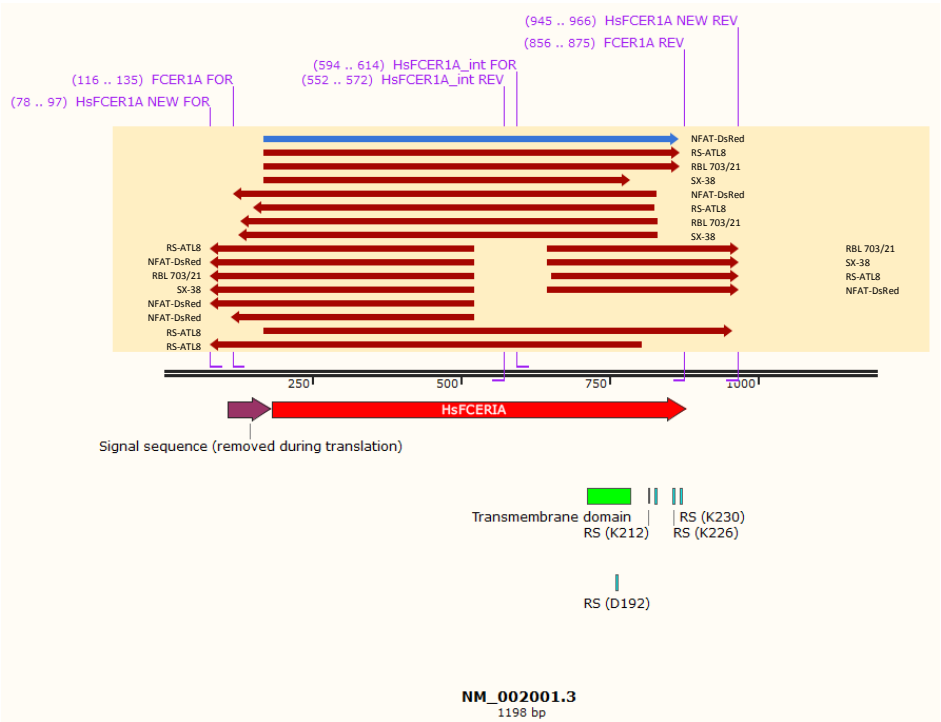

B

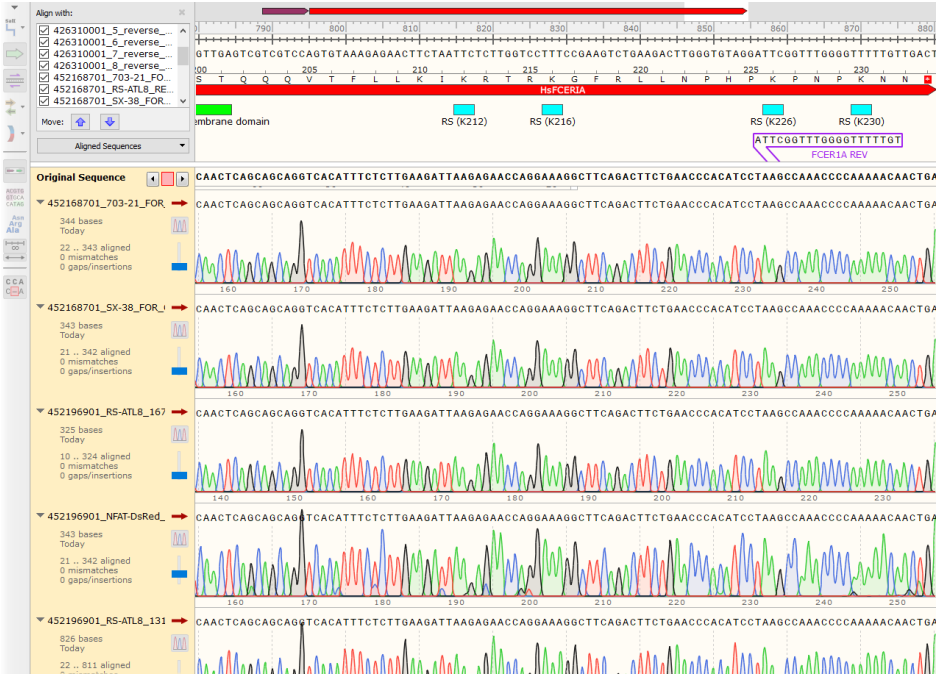

Supplement: S2 Fig — A) Map (SnapGene) of the coverage obtained from sequencing the cDNA with 6 different primers. The binding positions and names of the primers are indicated at the top of the map in purple. The bottom of the map also indicates the positions of the signal sequence peptide, the transmembrane domain and the 5 ER retention signals (RS) D192, K212, K216, K226 and K230. Full multiple coverage was obtained for all four humanized RBL cell lines, demonstrating complete identity of the cDNA sequences. B) Details of the chromatograms in the region containing the five known retention signals. (PDF) [file pone.0221034.s002.pdf]

**S3 Fig**

**A**

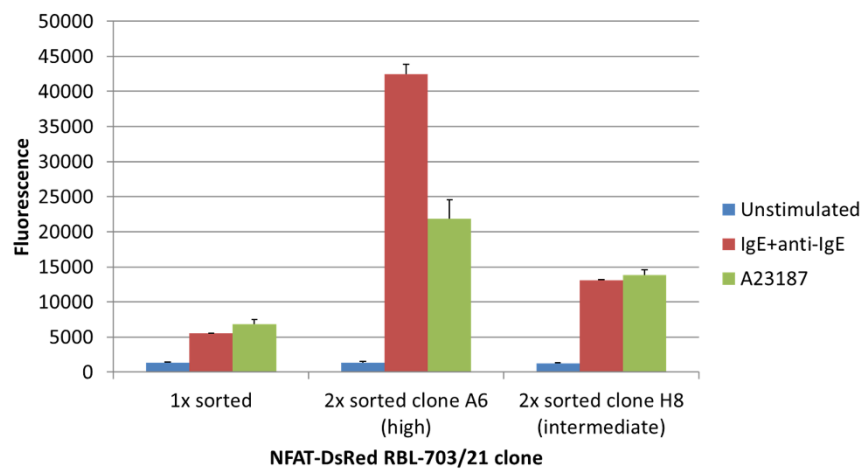

**B**

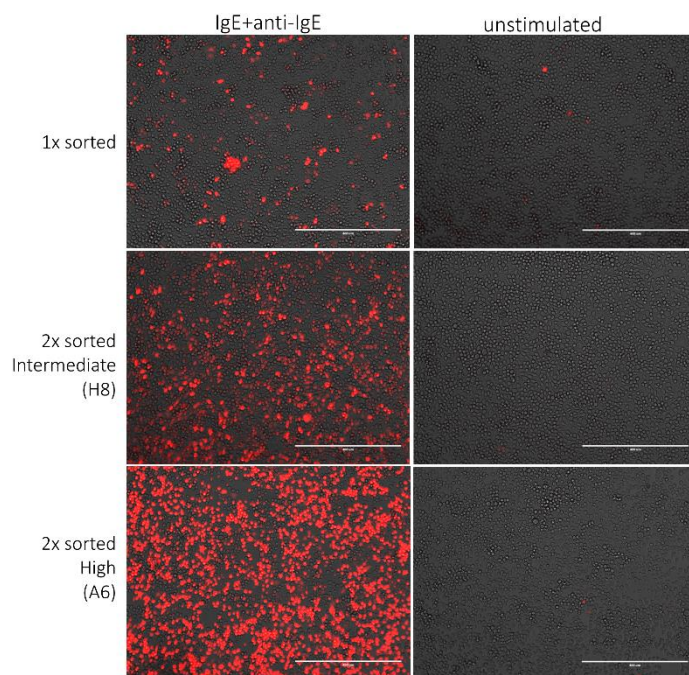

Supplement: S3 Fig — After a further incubation of 16–18 hours, responding cells producing DsRed were sorted by flow cytometry as single cells into 96-well plates, and clones allowed to grow and expand for several weeks. The highest responding cells were pooled and the process, consisting of activation, sorting and cloning was repeated once more. Individual 2x sorted clones were expanded and tested for their response to anti-IgE. A) shows the response of the 1x sorted cells, a 2x sorted high responding clone (A6) and an intermediate responding clone (H8) to activation via the IgE receptor (2 μg/mL anti-IgE) or 1μg/mL A23187. After removal of the medium, cells were lysed in 1% v/v Triton X-100 in DPBS and the lysate transferred to low-autofluorescence black plates. Fluorescence was read in an Infinite M200 plate reader (Tecan, Männedorf, Switzerland), using 530nm excitation and 590nm emission filters (this gave better results than the reported optimal 554nm excitation and 591nm emission for DsRed-Express2). B) shows the same cells with and without IgE-dependent activation under the EVOS fl microscope at 100x magnification using the RFP light cube. (PDF) [file pone.0221034.s003.pdf]
